# The Crossover Effects of Visuomotor Task Complexity in Training Reactive Agility of Ball Sports Athletes

**DOI:** 10.5114/jhk/210502

**Published:** 2025-11-20

**Authors:** Keyi Zhang, Wing Shan Chan, Hei Shuen Lau, Dongxiang Huang, Daniel Hung Kay Chow

**Affiliations:** 1 Department of Health and Physical Education, The Education University of Hong Kong, Hong Kong, China.; 2 School of Physical Education, Shaoguan University, Guangdong, China.

**Keywords:** visuomotor training, skill transfer, reaction time, team sport athletes, sport skills

## Abstract

Visuomotor reaction is a pivotal skill for athletes in ball sports. Training of such ability involves complex processing and coordination between cognitive functions and motor execution. Given the scattered literature on the topic related to task complexity, our study aimed to investigate the skill transfer effect among visuomotor tasks with different levels of complexity. Twenty-eight amateur ball players, with the mean age of 22.4 years old (SD = 1.9), were recruited and randomly assigned to either a simple or a complex visuomotor task intervention group, comprising bi-directional and multi-directional visuomotor training, respectively. Our study involved a four-week visuomotor agility training program. Visuomotor reaction times were recorded and analysed before and after the four-week intervention. The results demonstrated that both simple (F = 73.912; p < 0.01; ηp^2^ = 0.745) and complex (F = 80.6; p < 0.001; ηp^2^ = 0.762) visuomotor training were effective in enhancing participants' visuomotor performance at both levels of task complexity. The crossover effect of complex visuomotor training resulted in substantial improvement in both simple and complex visuomotor reaction time, suggesting that implementing complex visuomotor training could be more effective than a simple visuomotor training approach. These findings demonstrate the transferable effects associated with complex visuomotor agility training, highlighting its potential to enhance reactive agility across different levels of task complexity.

## Introduction

Executive function (EF) is suggested to be a key component of motor performance. It consists of higher- and lower-order processing. Higher-order functions control decision-making, anticipation and problem-solving abilities, while lower-order functions determine inhibitory control, cognitive flexibility and working memory. In team sports, higher-order executive functions are more needed because the gaming conditions require more complex mental processing to coordinate a series of emotional, cognitive and motor responses (Brimmell et al., 2022; Büchel et al., 2022; Diamond, 2013; Hagyard et al., 2021; Scharfen and Memmert, 2021). Agility, characterised as rapid whole-body movements in response to stimuli, involves motor components such as change of velocity and direction and cognitive components such as perception and decision-making (Young et al., 2015). Due to the overlapping neural mechanism, cognitive abilities of executive function contribute to the quality of agility performance (Brimmell et al., 2022).

Reactive agility, together with change-of-direction ability, comprises agility. It is critical in dynamic ball sports due to its reliance on quick visuomotor reactions to external cues (Popowczak et al., 2020; Zwierko et al., 2024b). Visuomotor reaction involves the ability to quickly translate visual information into precise muscle movements. Rapid visuomotor reactions determine players’ agility and on-field performance (Padrón-Cabo et al., 2020; Popowczak et al., 2020; Sheppard and Young, 2006; Zwierko et al., 2024b). Training that sharpens athletes’ ability to react swiftly to visual cues can significantly improve their performance (Popowczak and Zwierko, 2025; Zemkova et al., 2025; Zwierko et al., 2024a). Several studies have demonstrated that such training not only enhances visuomotor reaction times, but also boosts overall agility, benefiting athletes in unpredictable game scenarios (Horváth et al., 2022; Hülsdünker et al., 2018; Popowczak et al., 2020; Stone et al., 2019). Study results from Sinkovic et al. (2023) indicated that the training programme focused on athletes’ movement speed combined with strength when reacting to external stimuli could significantly improve their reactive agility and overall game performance as well. Therefore, training involving motor and perception components of reactive agility can benefit athletes’ overall reactive agility performance.

Practice-to-transfer is one of the goals of training that creates sufficient difficulties in task complexity to match game demands (Hodges and Lohse, 2022). As such, exploring the transfer of visuomotor skills among tasks of varying complexity levels could provide valuable insights for sports training and skill development.

Prior research has examined visuomotor reaction time by manipulating perceptual elements using eyewear equipment (Hülsdünker et al., 2021; Zwierko et al., 2024b) and exploring task complexity (Bootsma et al., 2018; Gutiérrez-Capote et al., 2024; Parrington et al., 2015). Investigations into how task complexity affects visuomotor reaction time have involved various approaches, including rule manipulation, auditory-visual stimuli, and body extremities. Gutiérrez-Capote et al. (2024) examined the impact of different task complexity on executive function by introducing rule restrictions during basketball training. Their findings indicated that altering task complexity influenced significantly athletes’ inhibitory capacity, a crucial aspect of lower-order executive function that regulates automatic responses and behavioural performance. While routine training can enhance inhibitory capacity for skill development in team sports, overly complex tasks may overwhelm processing capacity, leading to reduced performance (Hodges and Lohse, 2022).

Numerous studies have investigated the relationship between task complexity and motor learning (Akizuki and Ohashi, 2015; Gutiérrez-Capote et al., 2023; Parrington et al., 2015; Shuggi et al., 2017). In particular, Parrington et al. (2015) studied task complexity alongside stimulus modality (auditory-only and visual-only stimuli) to assess athletes’ executive function and response times in a laboratory setting. Their results demonstrated that task complexity notably affected participants’ perceptual reaction and motor response times, with auditory stimuli yielding faster executive reactions than visual stimuli. The study highlighted how increased task complexity primarily impacted cognitive processing rather than motor actions. Additionally, it revealed that visual stimuli could interfere with decision response times, underscoring the importance of visuomotor training in enhancing on-field performance, particularly in sports environments.

Engeroff et al. (2019) delved into the crossover and perceptual-cognitive effects of upper and lower extremity training on participants’ visuomotor reaction time using a computerised device. They found that lower extremity training enhanced reaction times for both lower and upper extremities, while upper extremity training did not produce the same effect. The absence of a crossover effect with upper extremity training was attributed to the task’s insufficient complexity to induce a training effect. Their results suggest that task difficulty is an important factor in training to achieve an observable outcome, aligning with the view of Hodges and Lohse (2022). However, their study was conducted in a laboratory setting using a computerised device measuring only partial body movement, consequently, it lacked comprehensiveness for accurate agility assessment or ecological validity.

The current study aimed to investigate the skill transfer effect among visuomotor tasks with different levels of complexity. The objectives of the study were as follows: 1) comparing the effects of simple and complex visuomotor training on participants’ reactive agility; 2) examining the crossover effect of simple and complex visuomotor training on complex and simple visuomotor reaction, respectively. This study also aimed to contribute to the development of effective agility training programs and protocols.

## Methods

### Power Analysis

To achieve a statistical power of 0.8 at an alpha error probability of 0.05, we calculated the required sample size with G*Power (version 3.1.9.4; Germany). Based on the whole-body average reaction time of pre- and post-training results reported by Wilkerson et al. (2020), Cohan’s F = 0.44 was calculated as our assumed effect size. The final calculation led to a required total sample size of twenty subjects; therefore, the final recruitment for our study resulted in twenty-eight participants, with 14 participants in each group.

### Participants

Twenty-eight participants, with a mean age of 22.4 ± 1.9 years, were randomly recruited for our study through advertisements on the university campus. Participants were team sports and racquet sports players who were categorised as tier 2 trained/developmental level following the framework of McKay et al. (2022). Participants were required to be healthy, physically active, and free from any acute or chronic physical illnesses and injuries (Table 1). Participants were instructed to abstain from consuming alcohol or caffeine and engaging in any physical activities 12 h prior to testing. However, they were permitted to continue their regular physical training, such as ball game matches, fitness training, and specific skill training related to their specific sports during the intervention sessions. The ethics committee of the Education University of Hong Kong, Hong Kong, China, granted ethical approval (approval code: HPE2022-23_H013; approval date: 04 July 2023), and participants provided informed consent before the experiment.

**Table 1:** Participants’ demographic information.

	Simple Visuomotor Intervention Group	Complex Visuomotor Intervention Group
Gender	10 Females / 4 Males	9 Females / 5 Males
Age (year)	22.3 ± 1.9	22.5 ± 2.1
Body height (m)	1.634 ± 0.071	1.638 ± 0.094
Body mass (kg)	56.5 ± 7.1	57.9 ± 9.8
Body mass index (kg/m^2^)	21.2 ± 2.2	21.4 ± 1.4

### Visuomotor Task Design

A kit of pods with electronic trackers was used to measure participants’ visuomotor reactions (BlazePod Trainer Pro kit, Blazepod Inc., Miami, Florida, United States). The kit incorporated LED light bulbs and proximity sensors that enabled the reception and transmission of energy upon touch. During testing, only one pod was illuminated at a time, which represented the visual stimuli. After a three-second countdown, participants were required to visually locate the illuminated pod, then run towards the pod and deactivate the light by tapping the sensor; this was considered one hit which represented the motor reaction. After successfully deactivating the light, the next light was illuminated. Jakobsen et al. (2011) reported in their study that reaction time was a valid measurement of cognitive ability, supported by a significant correlation.

**Figure 1:**
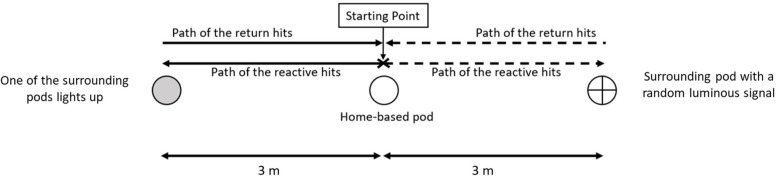
Setup of the simple reactive agility test.

Two visuomotor reaction tasks were designed with different levels of complexity: a simple visuomotor task and a complex visuomotor task. The simple visuomotor task involved two sources of visual stimuli, with three cones, each attached to a pod, arranged in a straight line. The central pod served as the home base, while the other two pods were positioned three metres apart on the right and left sides of the home base (Figure 1). The reliability of this test was moderate (ICC = 0.68).

**Figure 2:**
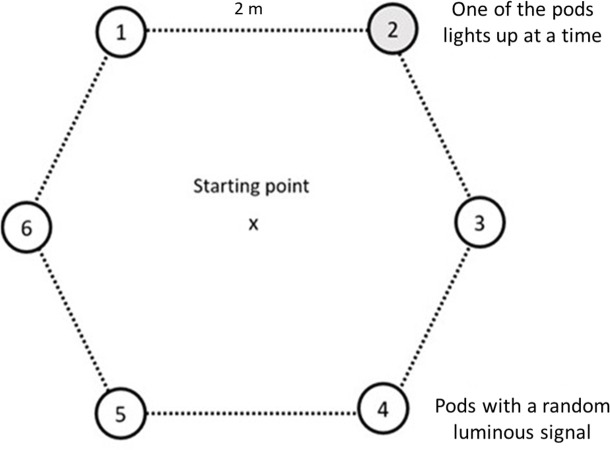
Setup of the complex reactive agility test.

The complex visuomotor task comprised six visual stimuli arranged in a regular hexagon shape. Six cones, each with attached pods, were spaced two meters apart from each other (Figure 2). Participants started at the centre of the hexagon layout, locating a stimulus after each hit until the end of each trial. The reliability of this test was also moderate (ICC = 0.55).

### Procedure

Prior to the tests, each participant completed a three-minute standardised warm-up consisting of high knees, leg swings (forward and backwards, lateral), truck twists, and lateral lunges. Participants were subjected to the reactive agility test, which involved both the simple visuomotor task and the complex visuomotor task. Each reactive agility test consisted of six trials in total, with three trials at each complexity level. To maintain task performance consistency among participants during the pre- and post-tests, three predetermined sets of lighting sequences were employed for both the simple and complex reactive agility tests. Each sequence was randomly assigned to illuminate a pod to prevent anticipation of the direction. By setting the lighting sequences beforehand, the total moving distance and the movement direction pattern for each trial remained consistent between the pre- and post-tests. The lighting sequences were not used during the intervention; instead, the training kit randomised the order of illumination. Each sequence ended automatically after the participant achieved a specific number of hits. In the simple reactive agility test, one sequence terminated after seven visuomotor reaction hits, while in the complex reactive agility test, each sequence automatically ended after the participant achieved eight visuomotor reaction hits.

After the reactive agility pre-test, participants were randomly assigned to either the simple visuomotor intervention group or the complex visuomotor intervention group for a four-week visuomotor reaction training intervention. The intervention comprised a total of eight sessions, with two training sessions per week (with at least 24 h in between). Each intervention session lasted 25 min and involved four sets of visuomotor reaction training tasks. The intervention training protocol mirrored the reactive agility test protocol, but omitted predefined lighting sequences. Instead, all visual stimuli were randomly activated by the training kit.

### Statistical Analysis

The average visuomotor reaction times for the last six hits in the simple reactive agility test and the final seven hits in the complex reactive agility test were recorded and calculated. Prior to conducting the two-way repeated measures analysis of variance (ANOVA), the assumptions of normality and homogeneity of variance were assessed. Normality was evaluated using the Shapiro-Wilk test, and homogeneity of variance was tested with the Levene’s test. Since all *p*-values exceeded 0.05, these results indicated that the assumptions were met. For each reactive agility test, ANOVA with mixed samples was conducted using statistical software (IBM SPSS, Version 28, USA), with time as the within-subject factor and the group as the between-subject factor. Effect sizes (ηp^2^) were reported for all significant findings, and post-hoc comparisons were carried out using the Fisher’s LSD test. Cohen’s *d* was calculated for the effect sizes of pairwise comparisons. A chi-square test was executed to confirm the comparability of age and gender between the two groups. The significance level was set at *p* = 0.05.

## Results

One dataset was excluded from the analysis because a participant in the simple visuomotor intervention group was found to be affected by caffeine during the data collection period. The baseline data from both the simple reactive agility test (*p* = 0.607) and the complex reactive agility test (*p* = 0.820) between the two training groups appeared to be comparable. The Shapiro-Wilk’s normality test was conducted on both groups. As all *p*-values were found to be greater than 0.05, the normality of the data was assumed.

**Figure 3:**
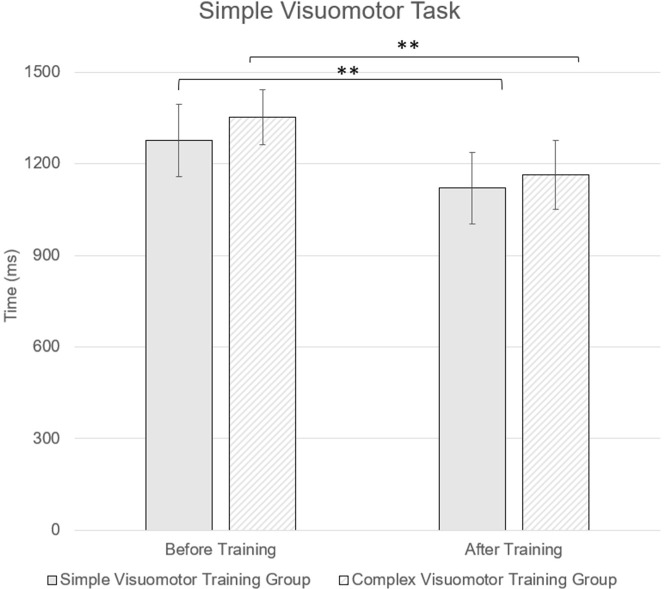
Comparison of the simple reactive agility test between simple and complex visuomotor intervention groups. ** indicates a significant difference at p < 0.001

In the simple reactive agility test, ANOVA revealed a significant time effect (F = 73.91; *p* < 0.01; ηp^2^ = 0.745). Further pairwise comparison measured with Cohen’s *d* revealed a large effect size in both simple (*d* = 1.517) and complex training groups (*d* = 1.771). Yet no significant interaction was found between the time and group factors (F = 0.64; *p* = 0.431; ηp^2^ = 0.025; *d* = 0.21) (Figure 3).

**Figure 4:**
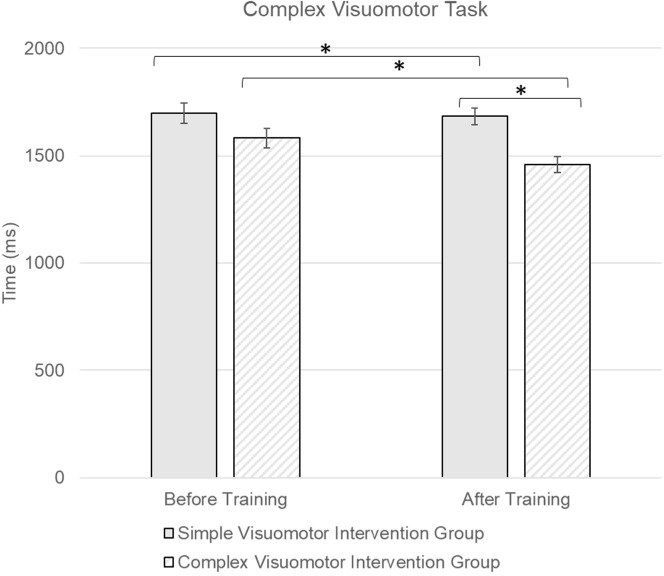
Comparison of the complex reactive agility test between simple and complex visuomotor intervention groups. * indicates a significant difference at p < 0.05

In the complex reactive agility test (Figure 4), ANOVA showed a significant time effect (F = 80.6; *p* < 0.001; ηp^2^ = 0.762), with Cohen’s *d* = 2.81 in the simple training group and *d* = 1.708 in the complex training group. Additionally, a significant interaction between the time and group factors was observed (F = 7.95; *p* = 0.009; ηp^2^ = 0.241; *d* = 0.815). The Fisher’s LSD post-hoc test further demonstrated a significant difference between the two intervention groups after training with a *p*-value of 0.032 and a medium effect size measured with Cohen’s *d* of 0.578.

## Discussion

The aim of this study was to investigate the skill transfer effect between simple and complex visuomotor training on reaction time. The lack of

group and testing time interaction from simple reactive agility test indicates that both simple and complex visuomotor interventions were effective at improving participants’ simple visuomotor reaction time and the complexity of the visuomotor training task did not have a significant effect on athletes’ simple visuomotor reaction time.

However, from the post hoc test result of the complex reactive agility test, the significant difference between the two intervention groups indicates that the complexity of visuomotor training tasks significantly affected athletes’ visuomotor reaction time in more complex reactive agility tasks. Even though the simple visuomotor intervention led to improved visuomotor reaction time in complex tasks, it was observed to be less effective than complex visuomotor task training.

The results confirmed crossover effects of visuomotor training on reactive agility and indicated that while simple visuomotor training led to enhanced complex visuomotor performance, this improvement was not as substantial as the one observed in the complex visuomotor training group. At the same time, the crossover effect of complex visuomotor training showed significant improvement in both simple and complex visuomotor reaction time. Our findings suggest that training effects of complex visuomotor tasks were more adequate in meeting various reactive agility demands under different game settings than simple visuomotor training.

The current results align with previous findings that evidenced training effects on visuomotor function, demonstrating that extensive training would lead to better performance of the trained area (Hülsdünker et al., 2018; Popowczak et al., 2020; Stone et al., 2019). Evidence has shown that visuomotor training influences the connectivity between the visual and motor cortex (Christiansen et al., 2020). In neuroscience studies, the enhancement of visual processing may be driven by cortical plasticity in the visual and visuomotor pathways, which continues to evolve due to experiences throughout the lifespan (Koch and Krenn, 2021). These findings not only support the significance of training effects on behavioural performance, but also suggest that skills can be transferred to other aspects of life or different contexts where the skills may be applied.

The enhancement of a ball game player’s simple visuomotor performance through complex training can be attributed to the development of perception and decision-making skills facilitated by increased task complexity. Poolton et al. (2006) suggested that higher task complexity imposed greater information processing load on individuals. In the context of complex training, ball game players are exposed to a substantial amount of information, requiring them to receive, process, and respond accordingly. This comprehensive training facilitates the development of cognitive domains, particularly the perceptual/decision-making components of agility (Zwierko et al., 2024b). When ball game players who have undergone such information-intensive training engage in a simple reactive agility task, the information processing load is significantly reduced compared to what they have been trained with. Consequently, their ability to respond to unanticipated stimuli becomes comparatively better. Therefore, complex training contributes to a greater extent to improved simple visuomotor reactive agility.

The task designs of simple and complex reactive agility tests vary based on the number and spatial arrangement of visual stimuli. The quantity of choices presented in the task significantly influences the level of task complexity. When participants are required to scan multiple options, it demands better visual attention in order to execute the accurate action; due to the operational nature of visual attention, the function requires multiple brain centres to act simultaneously in order to select the relevant information and filter out the irrelevant information. Such processing increases cognitive demand across different sectors of the cerebral cortex. Once participants identify the proper visual stimuli, the sensory information needs to be transferred into accurate motor responses quickly, which increases the individuals’ cognitive load, stimulating the perceptual-cognitive components of reactive agility.

Additionally, the arrangement of visual stimuli resulted in different moving ranges between the two tasks, requiring participants to plan and execute distinct motor reactions. In the simple visuomotor task, participants had to react to one of the two visual stimuli from the centre pod and return to it after each response. Each of the two sources of visual stimuli had an equal 50% chance of providing visual information. This task demanded lower-level visuomotor reactions to visual stimuli from limited lateral directions, restricting body movement and head-turning to lateral motions, thus placing lower demands on motor responses, decision-making, and information processing. In contrast, the complex visuomotor task required participants to exhibit a higher level of visuomotor response to stimuli from six different directions at the vertices of the surrounding hexagon. Each pod had a 17% chance of providing visual information due to the increased number of stimuli. The placement of stimuli prompted participants to move in various directions and incorporate additional head-turning movements to locate visual stimuli around them, demanding more extensive motor reactions and a wider range of movement.

The heightened task complexity placed increased demands on athletes' executive function, which was cultivated through the motor and perceptual components of the complex intervention. In the complex intervention, the presence of multiple options of potential stimuli required athletes to be more attentive and focused to execute more precise motor responses, thereby enhancing and sharpening their cognitive capacity and decision-making ability. This crucial aspect was lacking in the simple visuomotor training intervention, leading to less effective complex visuomotor reaction times in the simple intervention group. As a result, participants in the complex intervention group, when engaging in reactive agility tests, demonstrated the ability to process visual information into motor commands swiftly and accurately, regardless of the complexity of the visuomotor tasks. This enhanced processing ability ultimately contributed to their improvement in reactive agility.

## Practical Implications

Based on the current findings, we suggest incorporating visuomotor training tasks with a small or a moderate increase in task design complexity when training reactive agility for team sports or ball sports. Previous studies have demonstrated that tasks that are either overly complex or insufficiently complex may not yield training effects or skill transfer, underscoring the significance of task complexity in training to achieve desired outcomes. In our study, transfer of training effects from complex visuomotor training enhanced both complex and simple visuomotor reaction, which underscores the efficacy of such interventions. By expanding the training regimen and introducing elevated levels of task complexity, practitioners can explore innovative pathways to enhance reactive agility performance in simple tasks.

## Limitations

This study has some limitations that should be acknowledged. The regular ball-game training of the participants was not monitored during the four-week training intervention. Therefore, the effects of regular training on visuomotor performance remain unknown. Additionally, the experimental design did not include specific measurements to assess the cognitive abilities of athletes in the pre-test, which limits the robustness of the investigation. This should be addressed in future studies. The moderate reliability of the test design suggests that it may be less robust compared to existing visuomotor test designs, indicating that further revisions are needed to improve its reliability. Furthermore, one important limitation of our study is that we did not investigate the retention of the visuomotor training effect over time. Given that time is a significant factor in both simple and complex training, examining whether the observed improvements in visuomotor reaction time and reactive agility were sustained over a longer period would provide valuable insights into the long-term benefits of the training protocol. This study tested only two tasks in the training intervention; future research could explore the effects of other types of training tasks on reaction time. Most importantly, existing evidence has demonstrated that cognitive ability varies among athletes in different sports (Heilmann et al., 2022; Krenn et al., 2018; Voss et al., 2010). Furthermore, Domaradzki et al. (2021) confirmed the association of the sport type with reactive agility. This body of evidence highlights the necessity of including the sport type as a variable in investigations to yield more accurate results, which is a factor our study did not account for. Therefore, future studies should consider the effect of sport types when examining the transfer effects of task complexity on reactive agility.

## Conclusions

The aim of this study was to investigate the skill transfer effect between two visuomotor tasks with different levels of complexity. Our results demonstrated that although both simple and complex visuomotor training interventions were effective in enhancing participants’ visuomotor performance with significant crossover effects, only complex visuomotor training showed substantial improvement in both simple and complex visuomotor performance. The findings suggest that implementing sufficient complex visuomotor training as part of the team sportstraining regime could be more effective in enhancing various levels of reactive agility performance. Future studies exploring the visuomotor skill transfer may offer insightful information for developing sports skills and training.
